# Supplementary material for: Global dissociation of the posterior amygdala from the rest of the brain during REM sleep
Source: Commun Biol. 2022 Nov 28;5:1306. doi: 10.1038/s42003-022-04257-0 (PMC9705305; doi:10.1038/s42003-022-04257-0)
Supplement: Supplementary file 2 — Supplementary Information-New [file 42003_2022_4257_MOESM2_ESM.pdf]

List of Supplementary Materials

Supplementary Figure 1: Experimental design: plane-by-plane monitoring of large-scale brain hemodynamics, local field potentials and behavior in the same animal.

Supplementary Figure 2: Details of brain regions and sub-regions imaged.

Supplementary Table 1: Name of regions/subregions - number of recordings and of episodes per recording plane.

Supplementary Figure 3: Example of location of LFP electrodes implantation in rat #2, in both the atlas and a slice.

Supplementary Figure 4: Distribution of cerebral blood volume across brain regions for the four vigilance states studied (QW: quiet wake, AW: active wake, NREMS: non-REM sleep, REMS: REM sleep).

Supplementary Figure 5: Mean Activation Maps during tonic REM (top) and phasic REM (bottom).

Supplementary Figure 6: Dynamics of three veins highlighted in a sagittal plane.

Supplementary Figure 7: Representation of the number of recordings used to calculate the average cross-correlation in the connectivity matrix.

Supplementary Figure 8: Connectivity matrix with the individual substructures of the amygdala and the representation of the number of recordings used to calculate the average cross-correlation in the connectivity matrix.

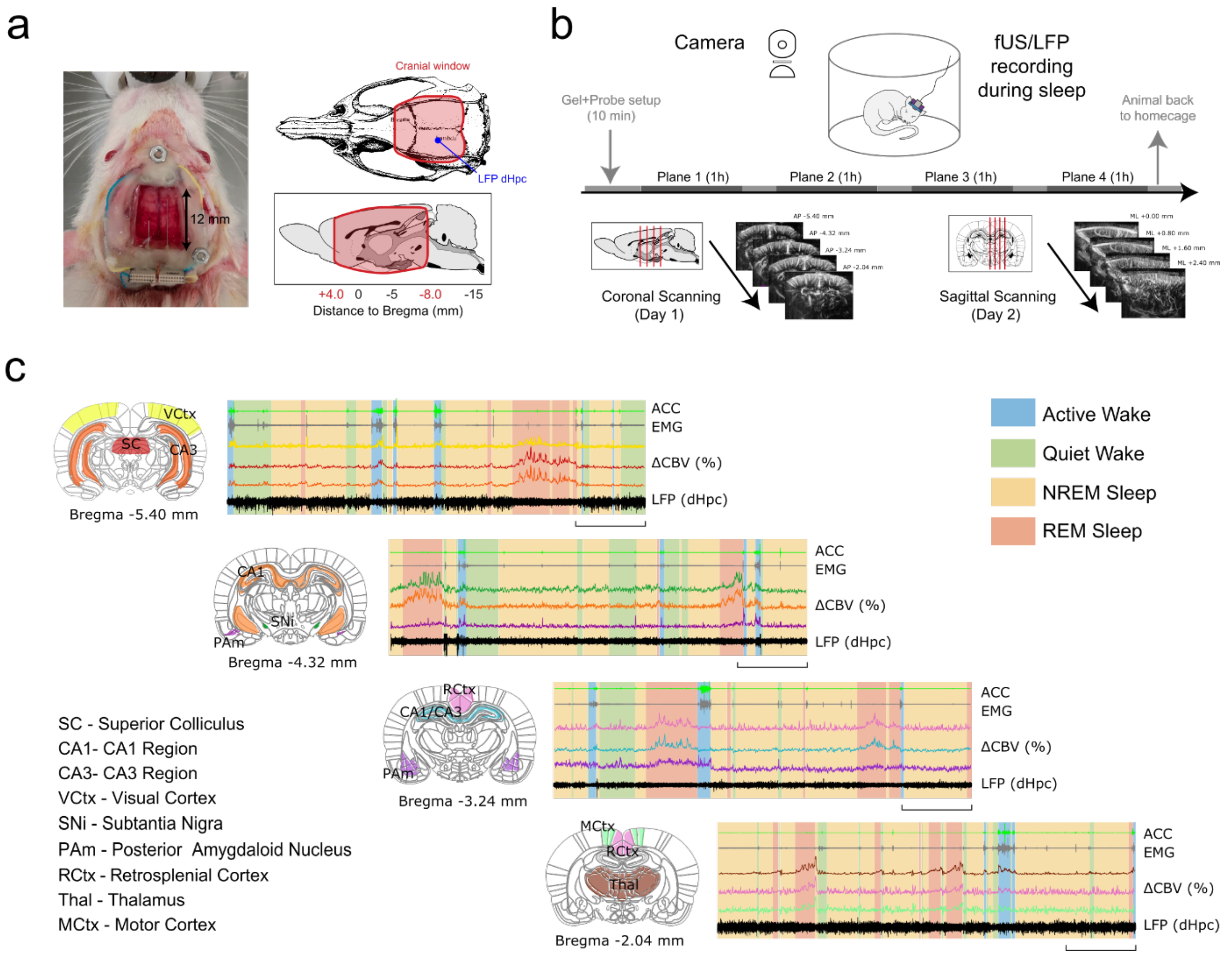

Supplementary Figure 1: Experimental design: plane-by-plane monitoring of large-scale brain hemodynamics, local field potentials and behavior in the same animal

a Top view of the fUS-LFP chronic surgical procedure. A large cranial window exposed the brain from Bregma AP +4.0 mm to Bregma AP +9.0 mm, which spanned approximately 3/4 of the total brain volume. LFP electrodes were implanted in one hemisphere underneath a sealed transparent polymer prosthesis. Schematics of the volume accessible to fUS imaging with LFP implantation site. **b** Details of the recording protocol. Animals were placed in a familiar recording chamber and the ultrasound probe was fixed to a wearable headstage (see Methods) without anesthesia. They underwent sequential coronal (or sagittal) brain scanning: 60 min per plane, 4-6 planes per day, interleaved with 20 min breaks, during which they slept spontaneously, before being placed back in their home cage. **c** Typical plane-by-plane coronal scanning over 4 different planes and associated temporal traces during a single recording day (green: accelerometer Y-axis, grey: EMG neck signal, black: LFP dorsal hippocampus, color: regional CBV traces). Each recording was sleep scored using EMG, accelerometer and LFP (see Methods) and divided into 4 phases: active wake (AW, blue), quiet wake (QW, green), non-REM sleep (NREMS, yellow) and REM sleep (REMS, red). Each plane was imaged for 1h and CBV traces are expressed in % change relative to a baseline set of frames (see Methods). Atlas images adapted from The Rat Brain in Stereotaxic Coordinates: Compact, Paxinos G. & Watson C., Copyright (2017), with permission from Elsevier.

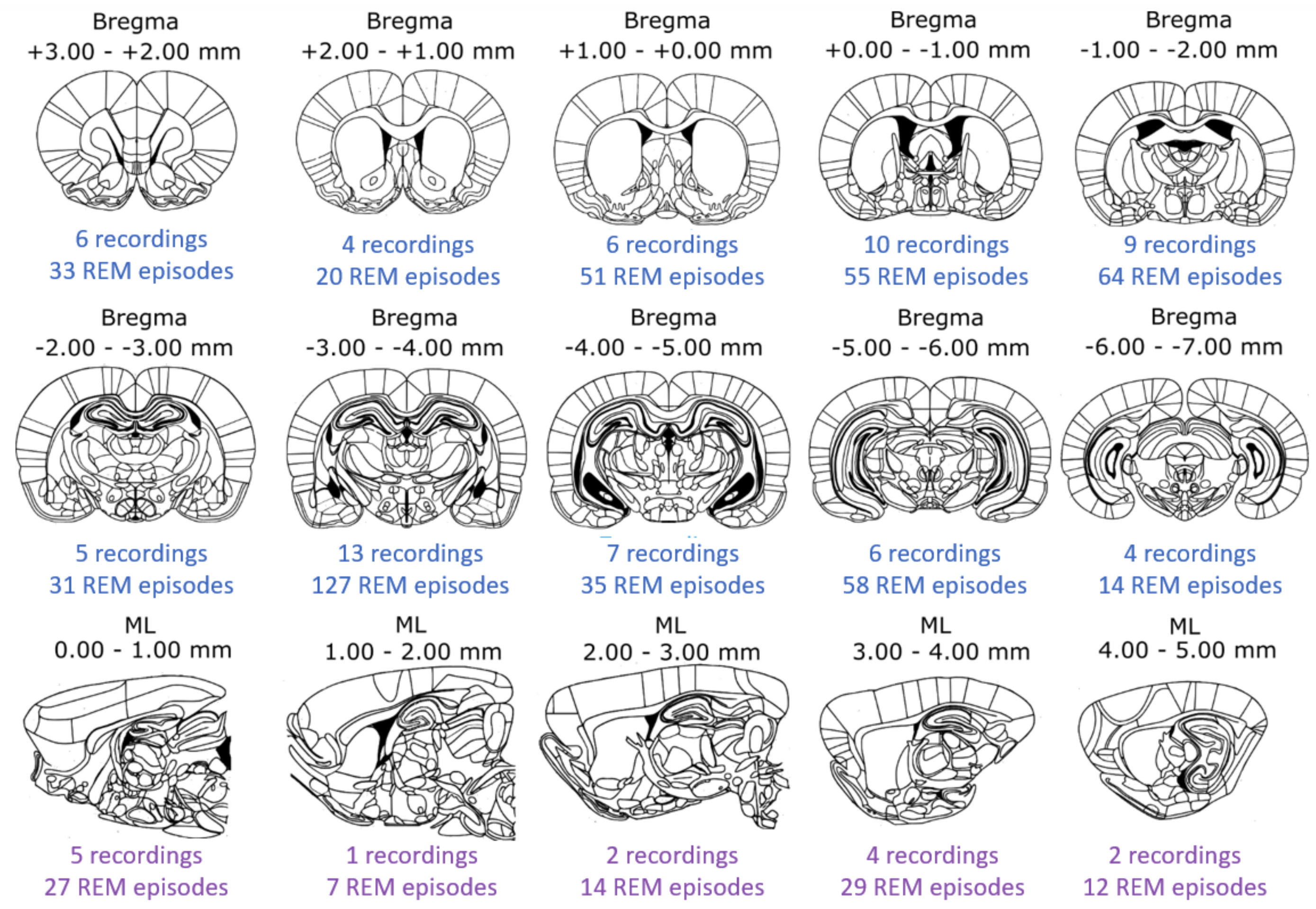

Supplementary Figure 2: Details of brain regions and sub-regions imaged.

Atlas images adapted from The Rat Brain in Stereotaxic Coordinates: Compact, Paxinos G. & Watson C., Copyright (2017), with permission from Elsevier.

		Plan span	Rats	Recordings	REM episodes	Important structures
Coronal plans	c1	β +3,0 mm – β +2,0 mm	2	6	33	Motor ctx, Cg ctx, Cpu
	c2	β +2,0 mm – β +1,0 mm	2	4	20	Motor ctx, Cg ctx, S1 ctx, Cpu
	c3	β +1,0 mm – β +0,0 mm	3	6	51	Motor ctx, Cg ctx, S1 ctx, CPu, MS
	c4	β +0,0 mm – β -1,0 mm	3	10	55	Motor ctx, Cg ctx, S1 ctx, CPu, GP, preoptic area
	c5	β -1,0 mm – β -2,0 mm	2	9	64	Motor ctx, RS ctx, S1 ctx, thalamus, anterior hypothalamus, amygdala
	c6	β -2,0 mm – β -3,0 mm	3	5	31	RS ctx, S1 ctx, dorsal hippocampus, thalamus, hypothalamus, amygdala
	c7	β -3,0 mm – β -4,0 mm	3	13	127	RS ctx, association ctx, Au ctx, dorsal hippocampus, thalamus, posterior hypothalamus, amygdala
	c8	β -4,0 mm – β -5,0 mm	3	7	35	RS ctx, visual ctx, Au ctx, dorsal & ventral hippocampus, thalamus, mammillary nucleus, PAG, amygdala
	c9	β -5,0 mm – β -6,0 mm	1	6	58	RS ctx, visual ctx, Au ctx, posterior hippocampus, SN, superior colliculus, PAG
	c10	β -6,0 mm – β -7,0 mm	1	4	14	RS ctx, visual ctx, Au ctx, enthorinal ctx, SN, superior colliculus, PAG
Sagittal plans	s1	ML +0,0 mm – ML +1,0 mm	2	5	27	Cg ctx, RS ctx, MS, superior colliculus, thalamus, preoptic area, hypothalamus, PAG
	s2	ML +1,0 mm – ML +2,0 mm	2	1	7	Motor ctx, association ctx, visual ctx, CPu, superior colliculus, thalamus, preoptic area, hypothalamus, hippocampus
	s3	ML +2,0 mm – ML +3,0 mm	2	2	14	Motor ctx, S1 ctx, visual ctx, CPu, thalamus, amygdala, hippocampus
	s4	ML +3,0 mm – ML +4,0 mm	2	4	29	Motor ctx, S1 ctx, visual ctx, CPu, GP, thalamus, amygdala, hippocampus
	s5	ML +4,0 mm – ML +5,0 mm	2	2	12	S1 ctx, visual ctx, CPu, GP, amygdala, hippocampus

Supplementary Table 1: Name of regions/subregions - number of recordings and of episodes per imaging plane.

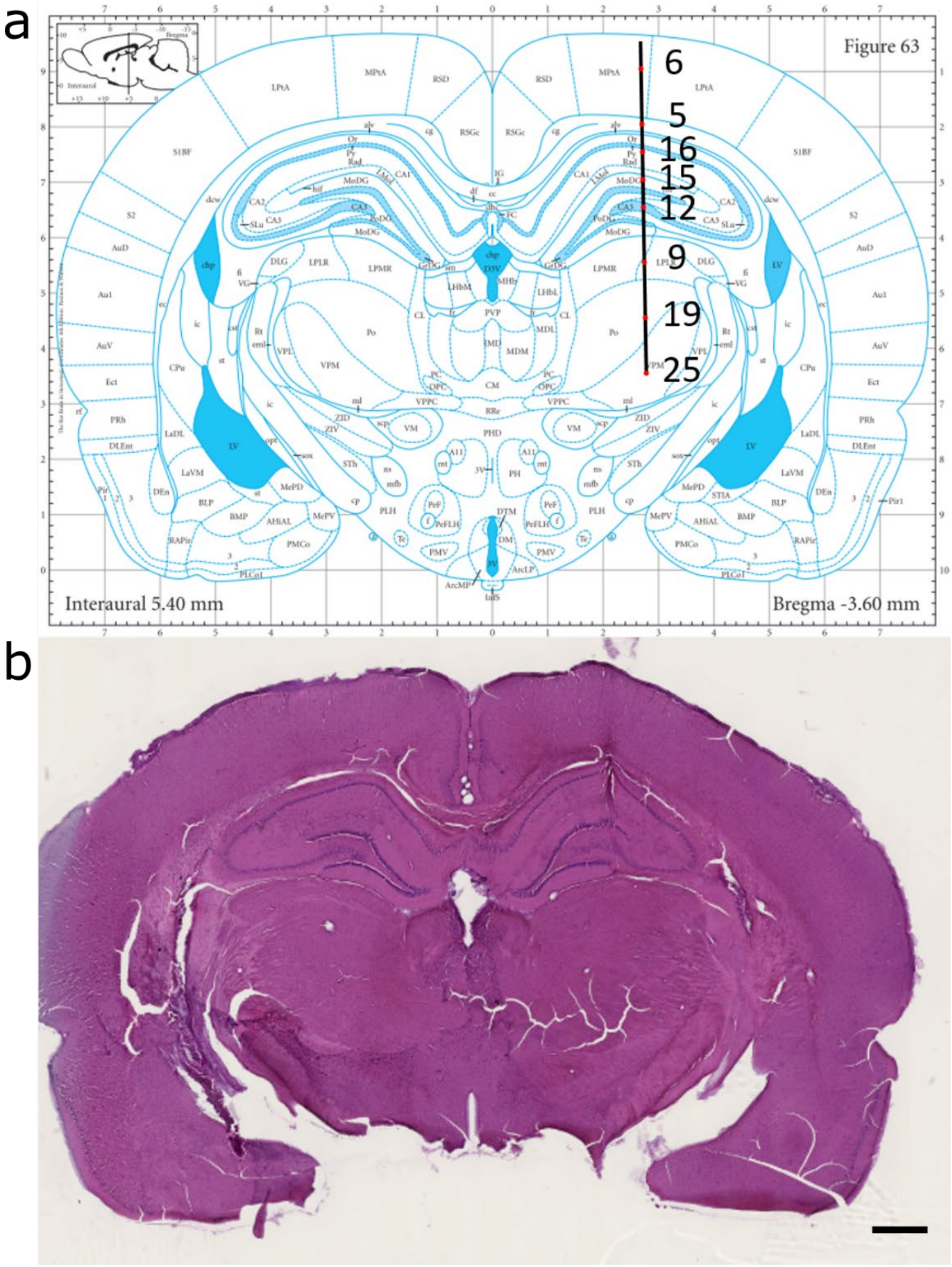

Supplementary Figure 3: Example of location of LFP electrodes implantation in rat #2 in both the atlas and a slice.

Image showing the location of electrodes **a** in the rat brain atlas (Paxinos and Watson, 2017). The brain slices were cut from fixed brains (paraformaldehyde 4%) using a vibratome and were colored using a mix of hematoxyline/eosine. We could see the track of the electrode. This track was then translated to the corresponding Paxinos plate and the recording points are placed knowing the distances between each of them. Sometimes the track was visible on multiple slices, we then used them all to define an average position of the electrode. Scale bar from **b** = 1mm. Atlas image reprinted from The Rat Brain in Stereotaxic Coordinates: Compact, Paxinos G. & Watson C., Copyright (2017), with permission from Elsevier.

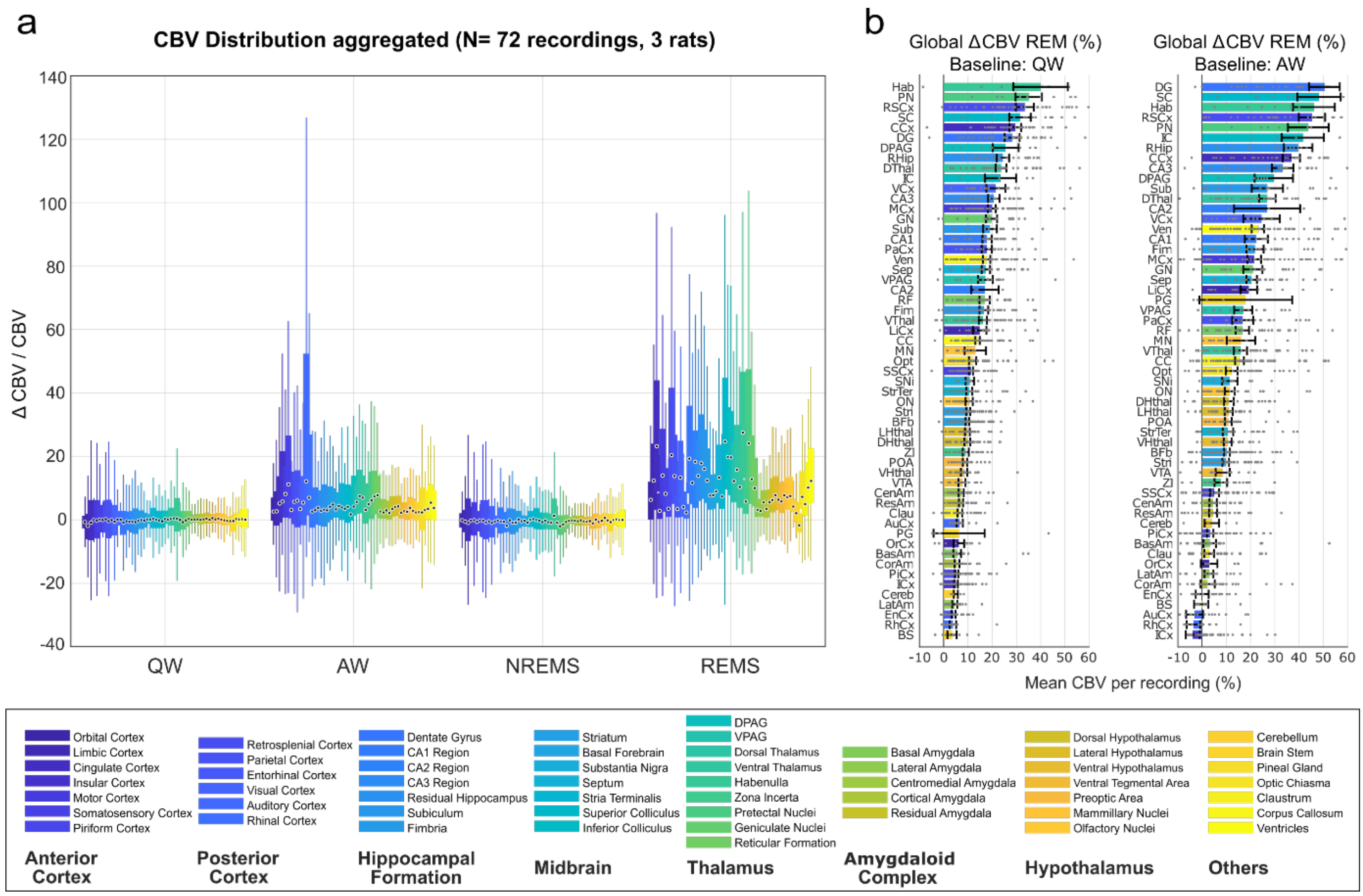

Supplementary Figure 4: Distribution of cerebral blood volume across brain regions for the four vigilance states studied (QW: quiet wake, AW: active wake, NREMS: non-REM sleep, REMS: REM sleep).

a Aggregated distributions of averaged CBV amplitude for the 4 different vigilance states (in 55 major brain regions for all coronal and sagittal recordings (N=3 animals, 72 recordings)), grouped by anterior cortical (dark blue, 7 regions), posterior cortical (blue, 6 regions), hippocampal (light blue, 7 regions), midbrain (cyan, 7 regions), thalamus (turquoise, 9 regions), amygdaloid complex (green, 5 regions), hypothalamus (orange, 7 regions) and others (yellow, 7 regions). The signal is expressed in delta CBV/CBV and the circles represent the means. QW and NREMS are characterized by a relative brain quiescence in all regions. AW is associated with increased cortical CBV levels, especially in the primary sensory areas. REMS is characterized by increased CBV in all brain regions with strongest effect in the hippocampal and limbic structures. The baseline was taken as the first consecutive 1-3 minutes of QW per recording **b** Mean CBV values during REM sleep for all brain regions sorted in descending amplitude of percentage. For each recording, we computed the mean value of the REMS distribution (grey dots) and then computed the mean across recordings (color bar) for all brain regions (Left: baseline taken as the first 1-3 min of QW (similar as **a**), Right: first 1-3 min of AW). Consistent with previous observations, hippocampus, retrosplenial cortex, superior colliculus and periaqueductal grey are robustly activated during REM sleep. This allows for the quantification of REMS hyperemia for all brain regions relative to QW and AW independently. Error bars denotes standard error of the mean.

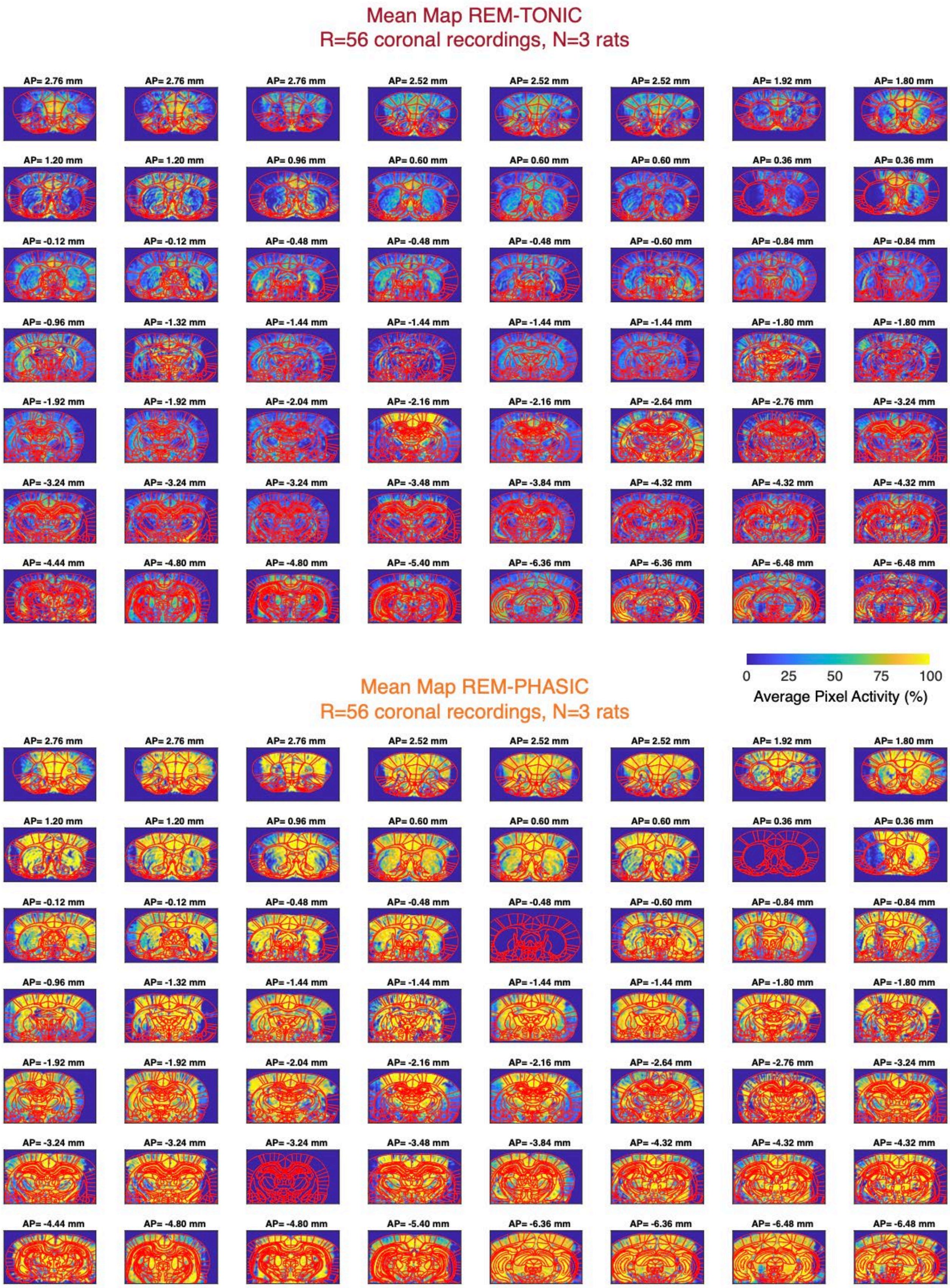

Supplementary Figure 5: Mean Activation Maps during tonic REM (top) and phasic REM (bottom) N=56 coronal recordings, sorted from anterior to posterior recording planes.

Atlas images reprinted from The Rat Brain in Stereotaxic Coordinates: Compact, Paxinos G. & Watson C., Copyright (2017), with permission from Elsevier.

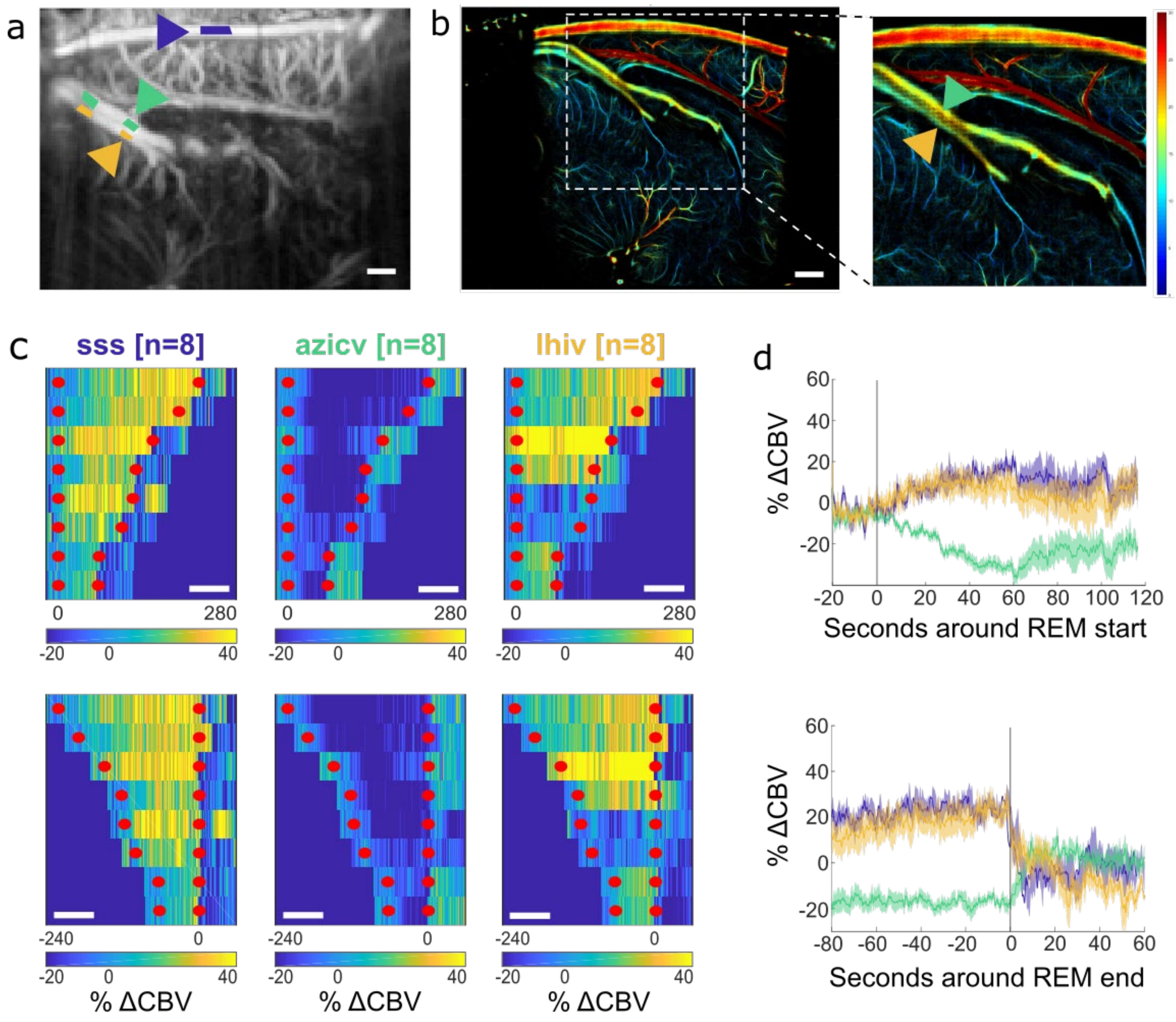

Supplementary Figure 6: Dynamics of three veins highlighted in a sagittal plane.

a Identification of the veins in sagittal plane using both ultrafast doppler images **a** and super-resoluted images **b**. These super-resoluted images, obtained following intravenous injections of microbulles were performed as previously described [71]. Due to the high contrast and excellent spatial resolution (10 μm), they allow separation of the azicv and the lhiv **b**. **c** Representation of all the REM episodes used to obtain the averaged values represented in **d** for each vessel, aligned either to the beginning of REM (top) or the end of REM episodes (bottom). Solid line represents the mean and shading represents the distribution. Beginning and end of REM episodes are shown with red dots **c** or vertical lines **e**. sss = superior sagittal sinus, azicv = azygos internal cerebral vein, lhiv = longitudinal hippocampal vein. Scale bar from **a, b** = 1 mm. Scale bars from **c** = 60 sec.

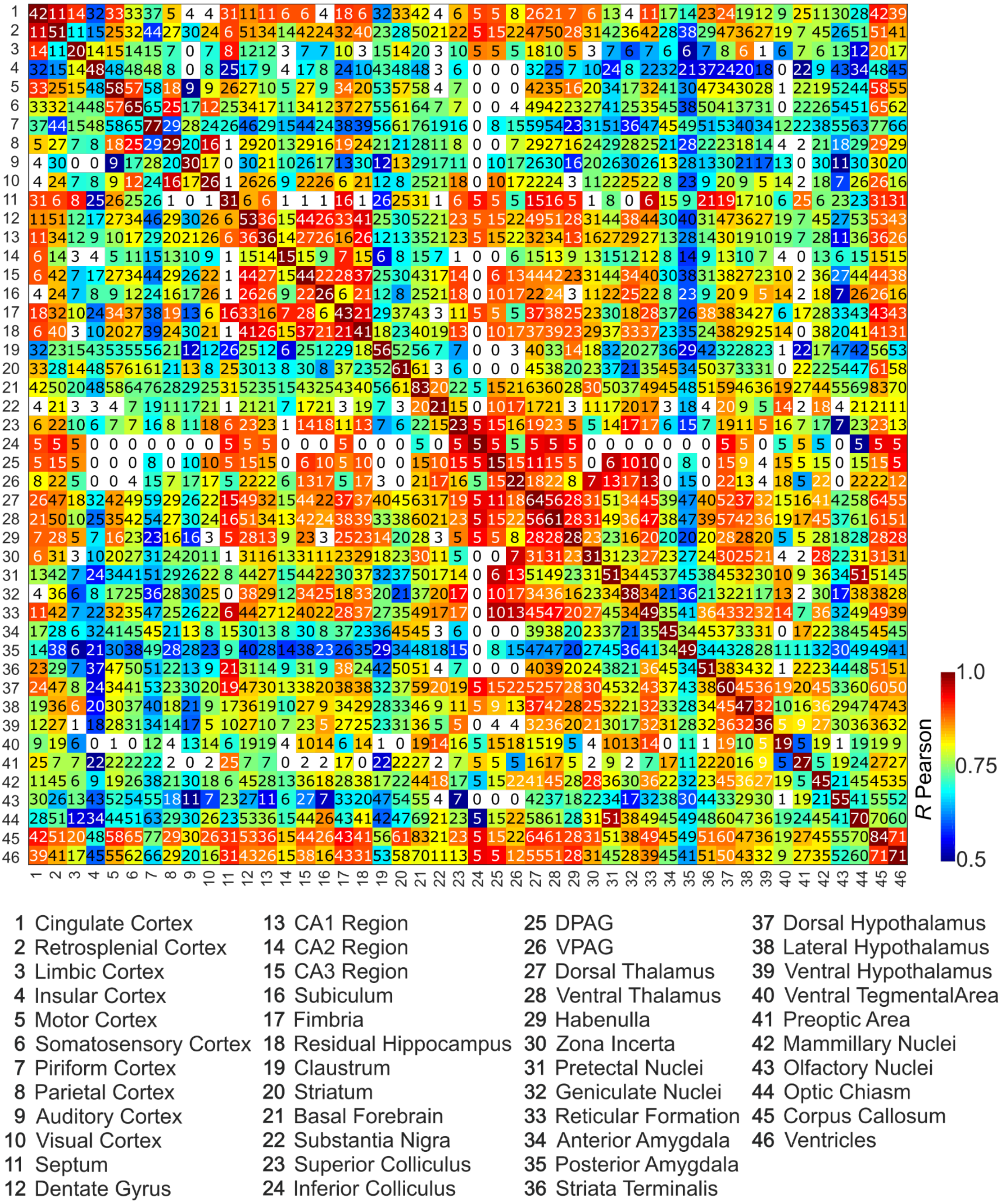

Supplementary Figure 7: Representation of the number of recordings used to calculate the average cross-correlation in the connectivity matrix.

Same connectivity matrix as presented in **Figure 5**, with a detail of the number of recordings used to average the cross-correlation values for each couple of regions. Couples with no value (not in the same plan) or with less than 5 recordings were left blank.

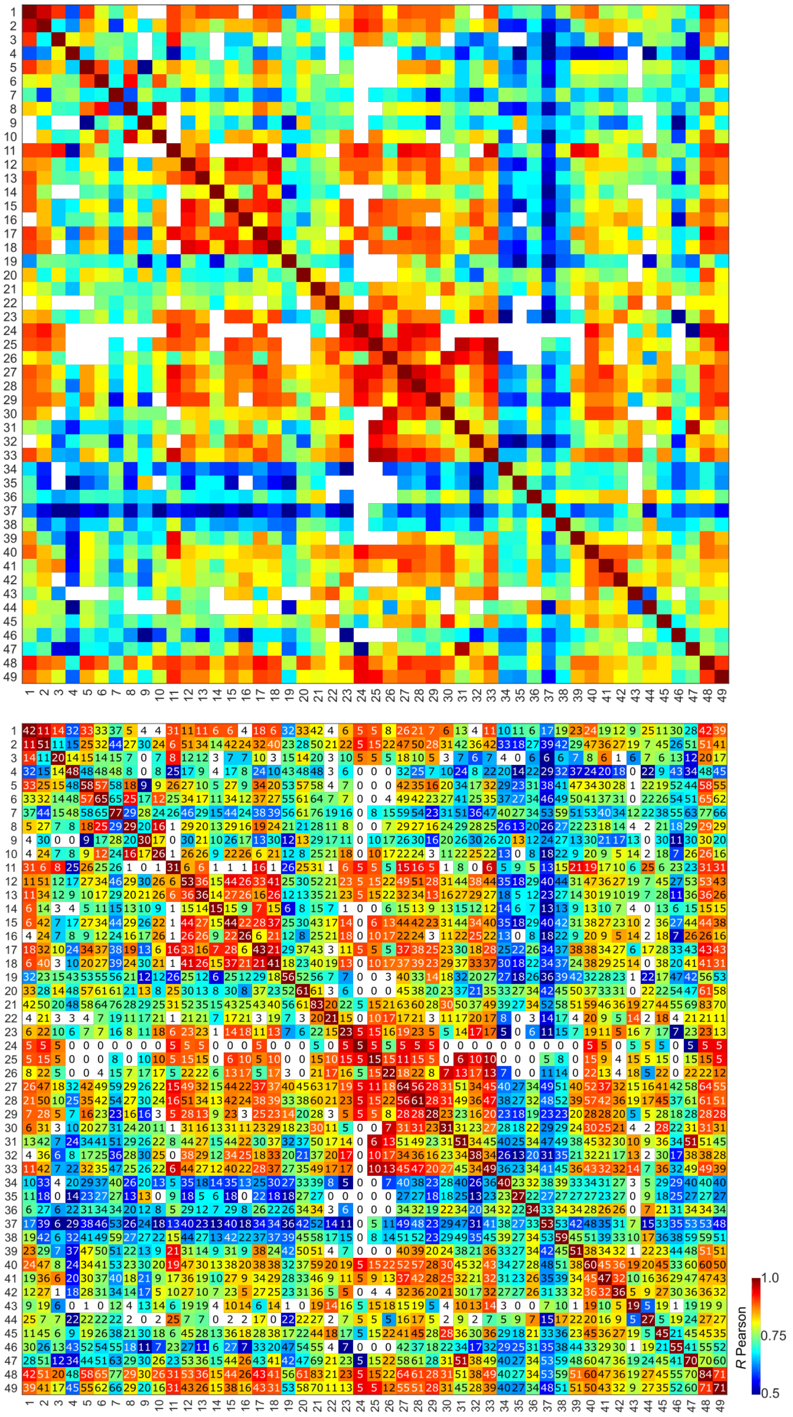

1 Cingulate Cortex
2 Retrosplenial Cortex
3 Limbic Cortex
4 Insular Cortex
5 Motor Cortex
6 Somatosensory Cortex
7 Piriform Cortex
8 Parietal Cortex
9 Auditory Cortex
10 Visual Cortex
11 Septum
12 Dentate Gyrus
13 CA1 Region
14 CA2 Region
15 CA3 Region
16 Subiculum
17 Fimbria
18 Residual Hippocampus
19 Claustrum
20 Striatum
21 Basal Forebrain
22 Substantia Nigra
23 Superior Colliculus
24 Inferior Colliculus
25 DPAG
26 VPAG
27 Dorsal Thalamus
28 Ventral Thalamus
29 Habenulla
30 Zona Incerta
31 Pretectal Nuclei
32 Geniculate Nuclei
33 Reticular Formation
34 Basal Amygdala
35 Lateral Amygdala
36 Centromedial Amygdala
37 Cortical Amygdala
38 Residual Amygdala
39 Striata Terminalis
40 Dorsal Hypothalamus
41 Lateral Hypothalamus
42 Ventral Hypothalamus
43 Ventral TegmentalArea
44 Preoptic Area
45 Mammillary Nuclei
46 Olfactory Nuclei
47 Optic Chiasm
48 Corpus Callosum
49 Ventricles

Supplementary Figure 8: Connectivity matrix with the individual substructures of the amygdala and the representation of the number of recordings used to calculate the average cross-correlation in the connectivity matrix.

Same connectivity matrix as presented in **Figure 5**, with a detail of the substructures of the amygdala and the number of recordings used to average the cross-correlation values for each couple of regions. Couples with no value (not in the same plan) or with less than 5 recordings were left blank.
