# Supplementary material for: Global dissociation of the posterior amygdala from the rest of the brain during REM sleep
Source: Commun Biol. 2022 Nov 28;5:1306. doi: 10.1038/s42003-022-04257-0 (PMC9705305; doi:10.1038/s42003-022-04257-0)
Supplement: Supplementary file 3 — Description of Additional Supplementary Files [file 42003_2022_4257_MOESM3_ESM.docx]

**Description of Additional Supplementary Files**

File name: Supplementary Movie 1

Description: A movie showing simultaneously the behavioral video from the IR camera, the Doppler film of the variations of CBV and the mean CBV trace during the REM episode shown in Figure 5b.

File name: Supplementary Data 1

Description: Detailed mean values of the CBV distributions in all regions across the different vigilance states.

File name: Supplementary Data 2

Description: Detailed correlation coefficients between the CBV in all regions and the REM index, the REM-PHASIC index and the REM-TONIC index.

File name: Supplementary Data 3

Description: Source data for figures 1c, 2d, 2e (with statistics), 3b, 3d and supplementary figure 4
